# Supplementary material for: Arthrocnemum Moq.: Unlocking Opportunities for Biosaline Agriculture and Improved Human Nutrition
Source: Plants (Basel). 2024 Feb 9;13(4):496. doi: 10.3390/plants13040496 (PMC10892625; doi:10.3390/plants13040496)
Supplement: Supplementary file 1 [file plants-13-00496-s001.zip › plants-2810169-supplementary.pdf]

Table S1. Material of *A. macrostachyum* and *A. meridionale* analyzed by ICP-MS: stems (ST) and seeds (S); SEM microscopy (numbers indicate the number of images taken per sample); SEM-EDX (number of analyzes performed by Energy Dispersive X-ray coupled to SEM); TEM microscopy (numbers indicate the number of images taken per sample) and X-ray diffraction (XR).

Taxa	Location	ICP Stems	ICP Seeds	SEM	SEM-EDX	TEM	XR
A. macrostachyum	Spain, Huelva, Ayamonte, “Salón de Santa Gadea”, 18.07.2018, 29SPB4218, UAM	-	-	14	20	-	-
A. macrostachyum	Spain: Huelva, Tinto river marshes, Moguer, 06.05.2005, 29SPB9028, UAM	ST1	-	-	-	-	X
A. macrostachyum	Spain: Huelva, Tinto river marshes, Moguer, 05.06.2005, 29SPB9028, UAM	ST2	-	-	-	-	-
A. macrostachyum	Spain: Huelva, Tinto river marshes, San Juan del Puerto, 27.06.2006, 29SPB9230, UAM	ST3	-	44	37 + Mapping	-	-
A. macrostachyum	Spain: Huelva, Tinto river marshes, San Juan del Puerto, 24.05.2006, 29SPB9230, UAM	-	-	31	24	-	-
A. macrostachyum	Spain: Huelva, Tinto river marshes, San Juan del Puerto, 07.08.2019, 29SPB9131, UAM	ST4	-	-	-	-	-
A. macrostachyum	Spain: Huelva, Tinto river marshes, Moguer, 27.09.2005, 29SPB9028, UAM	ST5	-	-	-	-	-
A. macrostachyum	Spain: Huelva, Tinto river marshes, La Rábida, 27.06.2006, 29SPB8320, UAM	ST6	-	-	-	-	-
A. macrostachyum	Spain: Huelva, Tinto river marshes, La Rábida, 17.07.2018, 29SPB8320, UAM	ST7	S2	14	15	-	-
A. macrostachyum	Spain: Huelva, Tinto river marshes, La Rábida, 17.07.2018, 29SPB8320, UAM	ST8	-	-	-	36	-
A. macrostachyum	Spain: Huelva, Tinto river marshes, La Rábida, 14.12.2017, 29SPB8320, UAM	-	-	17	3	113	-
A. macrostachyum	Spain: Huelva, Tinto river marshes, estuary, 17.07.2018, 29SPB8220, UAM	ST9	S1	-	-	77	X
A. macrostachyum	Spain: Huelva, Tinto river marshes, estuary, 27.06.2006, 29SPB8220, UAM	ST10	-	-	-	-	X
A. macrostachyum	Spain: Huelva, Tinto river marshes, estuary, 17.07.2018, 29SPB8220, UAM	ST11	-	-	-	-	-
A. macrostachyum	Spain: Toledo, Villasequilla de Yepes, 03.03.2019, 30SVK3520, UAM	ST12	-	-	-	-	-
A. macrostachyum	Spain: Toledo, Villasequilla de Yepes, 26.05.2009, 30SVK3814, MAF178626	-	-	26	25	-	-
A. macrostachyum	Spain: Toledo, Lillo, Laguna de Longar, 26.05.2009, 30SVJ7294, MAF178627	ST13	-	-	-	-	-
A. macrostachyum	Spain: Alicante, Urbanova, N-332, km 98, 07-10-2021, 30SYH1641, UAM	ST14	-	-	-	-	-
A. macrostachyum	Spain: Alicante, Calpe, Laguna de Ifach, 28.08.2015, 31SBC4579, MAF181282	ST15	-	-	-	-	-
A. macrostachyum	Spain: Murcia, San Pedro del Pinatar, Cotorillo marshes, 22.09.2009, 30SXG9692, MAF178628	ST16	-	-	-	-	-
A. macrostachyum	Spain: Castellón, Torreblanca, Parque Natural Cabanes, 28.09.2009, 31TBE6454, MAF178634	ST17	-	6	23	-	-
A. macrostachyum	Spain: Albacete, Cordovilla, Fuente García, 07.02.2019, 30SXH2166, MAF181294	-	S3	15	12	-	-
A. macrostachyum	Spain: Huelva, Tinto river marshes, estuary, 14.12.2017, 29SPB8220, UAM	-	S4	-	-	-	-
A. macrostachyum	Spain: Huelva, Tinto river marshes, La Rábida, 15.12.2017, 29SPB8320, UAM	-	S5	-	-	-	-
A. macrostachyum	Spain: Alicante, Santa Pola, salinas del Bras del Port, 02.09.2008, 30SYH0827, MAF178625	-	-	16	16	-	-
A. macrostachyum	Spain: Zaragoza, Belchite, El Planerón, 19.01.2017, 30TXL9782, MAF178638	-	-	2	5	-	-
A. macrostachyum	Spain: Islas Canarias, Fuerteventura, La Oliva, isla de Lobos, faro, 19.07.2022, 28RFS1582, UAM	-	-	16	16	-	-
A. macrostachyum	Spain: Islas Canarias, Lanzarote, Haría, Órzola, playa del Caletón Blanco, 21.07.2022, 28RFT5133, UAM	-	-	3	3	-	-
A. meridionale	Tunisia: Gobernación de Tozeur, Chott el Djerid, end of the lake, km 19 Tozeur, 08.12.2019, 32SMC3561, UAM	ST18	-	-	-	-	-
A. meridionale	Tunisia: Gobernación de Sfax, Sidi Mansour, cerca de Khalij, 03.12.2019, 32SPD6549, UAM	ST19	-	-	-	-	-
A. meridionale	Malta: San Pawl il-Baħa, Salina Nature Reserve, on rocky ground, 16.08.2020, 33SVV4878, MAF178645	ST20	-	-	-	-	-
A. meridionale	Tunisia: Sfax, Kerkennah Island, Chergui, Kraten marshes, 17.10.2019, 32SQD0554, MAF180599	-	S6	-	-	-	-

A. meridionale	Tunisia: Gobernación de Sfax, Sidi Mansour, cerca de Khalij, 03.12.2019, 32SPD6549, UAM	-	-	33	33	-	
A. meridionale	Italy: Sardinia, Isle Sant'Antioco, Is Pruinis-Playa, 29.11.2012, 32SMJ5321, MAF178642	-	S7	-	-	-	-
A. meridionale	Italy: Sicily, Siracusa, Riserva Naturale Orientata Oasi Faunistica di Vendicari, 16.06.2017, 33SWA0974, MAF178639	-	-	16	-	-	-
A. meridionale	Italy, Sicily, Saline di Trapani e Paceco nature reserve, 30STC8207, 14.06.2017, MAF178641	-	-	16	3	-	-
A. meridionale	Italy: Sicily, Palermo, Cinisi,Cinisi beach, 15.06.2017, 33SUC3225, MAF178640	-	-	32	-	-	-

Table S2. ICP-MS (ppm) of succulent stems of *A. macrostachyum* (triplicates). Symbology: M (mean) and SD (Standard deviation). Samples nomenclature referred in Table S1.

**ID**	**Na**	**Mg**	**K**	**Ca**	**Fe**	**Zn**	**B**	**Mn**	**Mo**	**Cu**	**Ni**	**Ba**	**Sr**	**Cr**	**As**	**Cd**	**Pb**
ST1	86994.19	5924.84	22502.63	2888.35	238.07	19.30	23.64	18.49	1.01	10.13	7.18	2.24	13.47	13.88	1.90	0.19	0.84
ST1	88961.74	6461.97	24953.92	3003.03	241.76	19.21	22.86	19.24	0.94	9.59	6.37	2.00	13.81	14.22	0.71	0.12	0.68
ST1	97794.25	6473.98	23039.47	3117.51	263.53	18.91	22.95	19.10	0.98	10.26	7.29	2.40	13.58	15.79	0.56	0.13	0.82
**M**	91250.06	6286.93	23498.67	3002.96	247.79	19.14	23.15	18.94	0.98	9.99	6.95	2.22	13.62	14.63	1.06	0.15	0.78
**SD**	5752.19	313.64	1288.54	114.58	13.76	0.20	0.43	0.40	0.04	0.35	0.50	0.20	0.17	1.02	0.73	0.04	0.09
ST2	121200.72	7394.56	33618.73	5276.00	78.62	36.64	29.57	8.25	1.31	6.31	0.88	0.80	38.84	7.03	0.00	0.20	0.26
ST2	126808.56	7150.62	33520.42	5358.40	81.47	34.71	32.32	8.08	1.29	5.85	0.96	0.80	38.25	7.14	0.00	0.21	0.23
ST2	112489.63	6720.59	34014.14	4963.94	73.25	34.32	35.59	7.36	1.26	5.62	0.76	0.73	36.20	6.55	0.00	0.18	0.23
**M**	120166.30	7088.59	33717.76	5199.45	77.78	35.22	32.50	7.89	1.29	5.92	0.87	0.77	37.76	6.90	0.00	0.20	0.24
**SD**	7215.29	341.24	261.33	208.07	4.18	1.24	3.01	0.47	0.03	0.35	0.10	0.04	1.39	0.31	0.00	0.02	0.02
ST3	53611.25	9125.04	15568.71	8108.88	427.50	28.80	63.83	13.38	0.67	28.28	3.39	3.99	65.88	6.67	2.23	0.29	3.27
ST3	69931.72	8492.80	14950.45	7372.24	424.30	25.11	57.13	12.50	0.45	26.56	3.60	4.13	59.35	7.05	2.12	0.36	3.06
ST3	75558.20	9509.43	15072.37	8833.26	495.88	28.92	63.97	14.24	0.54	30.97	4.46	4.87	68.36	8.32	2.50	0.38	3.31
**M**	66367.06	9042.43	15197.18	8104.79	449.23	27.61	61.64	13.37	0.55	28.61	3.82	4.33	64.53	7.35	2.29	0.34	3.21
**SD**	11399.44	513.33	327.48	730.52	40.43	2.17	3.91	0.87	0.11	2.22	0.57	0.47	4.65	0.86	0.19	0.05	0.13
ST4	121620.37	6438.00	26553.07	6451.28	250.28	17.59	31.75	24.17	0.98	11.97	0.47	4.22	24.13	1.23	1.34	0.44	2.39
ST4	121664.44	6297.30	28085.13	6451.07	246.51	15.92	34.07	24.40	1.06	12.68	0.23	4.93	25.05	0.44	1.27	0.39	2.36
ST4	125620.13	6249.79	26671.75	6691.78	233.47	15.16	35.12	23.83	0.96	11.18	0.40	5.42	22.89	0.51	1.22	0.38	2.31
**M**	122968.31	6328.36	27103.31	6531.38	243.42	16.22	33.65	24.13	1.00	11.94	0.37	4.86	24.02	0.73	1.28	0.40	2.35
**SD**	2296.64	97.88	852.34	138.91	8.82	1.24	1.73	0.28	0.05	0.75	0.12	0.60	1.08	0.44	0.06	0.03	0.04
ST5	117867.17	7729.87	5387.12	7819.29	704.18	37.04	44.64	20.82	1.39	33.73	15.32	8.39	26.12	29.56	3.95	1.85	8.18
ST5	101109.56	7489.71	7116.72	8156.53	567.32	34.44	50.62	17.83	1.49	33.59	11.94	8.23	28.41	21.90	2.82	1.43	5.95
ST5	123497.48	8032.30	5947.78	8513.12	740.46	40.53	48.58	22.48	1.51	36.58	16.87	9.65	28.27	32.45	4.01	1.67	8.63
**M**	114158.07	7750.63	6150.54	8162.98	670.65	37.34	47.95	20.38	1.46	34.64	14.71	8.76	27.60	27.97	3.59	1.65	7.59
**SD**	11645.72	271.89	882.45	346.96	91.31	3.05	3.04	2.36	0.06	1.69	2.52	0.78	1.28	5.45	0.67	0.21	1.43
ST6	69261.02	8409.76	23723.88	3854.36	292.03	33.48	49.36	10.60	2.33	49.10	3.10	3.52	49.22	5.31	0.96	0.12	2.08
ST6	78578.09	4748.03	13620.92	2491.51	169.35	19.61	28.58	6.08	1.48	29.23	0.06	2.30	29.08	2.75	0.35	0.04	1.32
ST6	85484.19	4988.73	12619.41	2307.09	166.48	19.46	27.26	6.06	1.46	28.20	0.11	2.15	27.32	2.95	0.29	0.03	1.40
**M**	77774.43	6048.84	16654.74	2884.32	209.29	24.18	35.07	7.58	1.76	35.51	1.09	2.66	35.21	3.67	0.53	0.06	1.60
**SD**	8141.39	2048.15	6142.50	845.12	71.67	8.05	12.40	2.62	0.50	11.78	1.74	0.75	12.17	1.42	0.37	0.05	0.42

ID	Na	Mg	K	Ca	Fe	Zn	B	Mn	Mo	Cu	Ni	Ba	Sr	Cr	As	Cd	Pb
ST7	58893.94	6139.17	10825.83	3452.88	334.80	18.90	44.45	74.49	1.38	53.44	4.51	2.88	39.34	9.66	0.95	0.08	3.73
ST7	53658.07	5564.07	9432.96	3287.33	291.02	18.60	49.66	68.51	1.23	49.58	4.32	2.47	36.09	8.39	1.20	0.08	3.21
ST7	61717.03	6390.67	10795.30	3636.69	325.27	20.34	43.83	78.04	1.38	55.55	4.88	2.93	41.47	9.77	1.10	0.08	3.45
**M**	58089.68	6031.30	10351.36	3458.96	317.03	19.28	45.98	73.68	1.33	52.85	4.57	2.76	38.97	9.27	1.09	0.08	3.46
**SD**	4089.23	423.72	795.50	174.76	23.03	0.93	3.20	4.82	0.09	3.02	0.29	0.25	2.71	0.77	0.13	0.00	0.26
ST8	49850.49	6684.52	13706.84	4610.99	276.33	26.86	37.77	25.35	1.51	53.03	2.98	2.58	30.95	4.86	0.81	0.14	2.95
ST8	60233.28	6087.81	10357.00	4143.72	272.79	22.90	38.05	22.56	1.64	49.86	2.45	2.37	25.57	4.41	0.00	0.00	3.81
ST8	63159.70	6304.62	11735.53	4286.17	251.67	20.12	34.96	22.39	1.80	47.16	2.17	2.66	28.40	3.52	0.00	0.00	3.80
**M**	57747.82	6358.98	11933.12	4346.96	266.93	23.29	36.93	23.44	1.65	50.01	2.53	2.54	28.31	4.26	0.27	0.05	3.52
**SD**	6994.06	302.04	1683.64	239.50	13.33	3.39	1.71	1.66	0.15	2.94	0.41	0.15	2.69	0.68	0.47	0.08	0.50
ST9	97134.04	9397.05	13876.90	4654.88	2816.42	135.02	83.78	83.72	13.02	89.27	7.86	11.60	44.47	16.70	18.97	0.31	50.49
ST9	71967.39	7729.78	15298.47	4405.98	2026.15	102.96	83.16	70.41	11.81	70.73	5.31	6.97	44.68	10.71	12.92	0.08	32.76
ST9	61007.19	6146.62	11591.20	3735.21	1564.22	83.11	62.48	55.39	9.90	57.13	4.19	6.99	34.43	9.40	7.22	0.00	32.69
**M**	76702.87	7757.82	13588.86	4265.36	2135.60	107.03	76.47	69.84	11.58	72.38	5.78	8.52	41.19	12.27	13.03	0.13	38.65
**SD**	18523.12	1625.39	1870.35	475.69	633.23	26.19	12.13	14.17	1.58	16.13	1.88	2.67	5.86	3.89	5.88	0.16	10.26
ST10	145029.84	6006.39	40187.19	4483.26	333.08	18.94	43.50	33.55	1.15	50.80	8.96	5.08	21.05	16.84	0.82	0.59	1.82
ST10	137899.34	5252.73	12935.86	3977.23	339.88	19.08	39.20	34.23	1.16	51.25	6.54	4.98	19.68	16.24	1.17	0.56	1.78
ST10	156656.68	4935.98	11274.64	3627.93	402.45	20.97	35.07	34.23	1.15	60.04	8.63	5.42	17.24	20.49	0.73	0.61	2.02
**M**	146528.62	5398.36	21465.90	4029.47	358.47	19.66	39.26	34.00	1.15	54.03	8.04	5.16	19.32	17.86	0.90	0.58	1.87
**SD**	9468.06	549.86	16234.38	430.05	38.24	1.14	4.22	0.39	0.01	5.21	1.32	0.23	1.93	2.30	0.23	0.03	0.13
ST11	62464.66	4676.37	16511.45	1783.15	334.83	25.42	27.41	6.45	1.15	68.83	1.89	5.44	22.14	3.97	1.10	0.00	4.21
ST11	60141.53	4559.73	15802.59	1808.31	288.15	26.63	24.19	5.93	1.23	71.82	1.91	4.80	20.49	3.96	1.40	0.00	4.04
ST11	65621.81	4983.61	16717.57	1898.01	336.05	26.26	30.29	6.38	1.34	73.57	1.99	5.09	22.30	4.94	0.82	0.00	4.65
**M**	62742.67	4739.91	16343.87	1829.82	319.68	26.10	27.30	6.25	1.24	71.40	1.93	5.11	21.64	4.29	1.11	0.00	4.30
**SD**	2750.70	218.97	479.96	60.38	27.31	0.62	3.06	0.28	0.10	2.40	0.05	0.32	1.01	0.56	0.29	0.00	0.31
ST12	296890.47	41357.39	43864.30	4887.65	450.76	77.99	428.97	172.64	10.19	45.89	13.97	2.46	40.82	8.94	0.44	0.26	0.23
ST12	323315.17	44174.65	49223.93	5706.97	428.88	83.50	370.69	181.88	10.81	46.70	15.32	2.34	42.03	9.82	0.46	0.25	0.21
ST12	313043.90	38942.15	43034.98	4772.19	374.64	77.73	392.24	166.54	10.02	44.08	13.71	2.08	36.72	8.57	0.31	0.22	0.18
**M**	311083.18	41491.40	45374.40	5122.27	418.10	79.74	397.30	173.69	10.34	45.56	14.33	2.29	39.86	9.11	0.41	0.24	0.21
**SD**	13321.02	2618.82	3359.48	509.64	39.19	3.26	29.47	7.72	0.42	1.34	0.86	0.19	2.79	0.64	0.08	0.02	0.03
ST13	236945.04	41369.16	64238.30	11784.80	397.09	40.41	12.75	75.37	3.78	25.60	8.32	5.48	96.21	10.73	0.41	0.97	0.42
ST13	251440.81	43498.67	65356.12	11835.92	412.39	40.23	0.00	81.10	3.30	25.26	8.71	4.45	94.98	11.09	0.27	0.98	0.49
ST13	230455.30	41112.74	65017.81	11830.70	391.48	41.09	1.53	76.92	3.36	25.43	10.28	10.21	89.97	9.81	0.17	1.00	0.42
**M**	239613.72	41993.52	64870.74	11817.14	400.32	40.58	4.76	77.80	3.48	25.43	9.11	6.71	93.72	10.54	0.28	0.98	0.45
**SD**	10744.27	1309.79	573.24	28.13	10.82	0.45	6.96	2.96	0.26	0.17	1.04	3.07	3.30	0.66	0.12	0.02	0.04
ST14	147548.74	13506.05	15703.60	8069.07	504.06	19.21	219.59	36.88	4.32	7.23	14.40	5.63	85.91	26.70	0.24	0.25	0.74
ST14	120320.76	11616.30	12561.17	6917.47	432.35	15.99	255.97	30.59	3.68	5.25	11.55	4.74	75.35	22.81	0.29	0.23	0.61
ST14	160904.70	16157.89	17747.69	9423.14	519.90	21.39	322.65	40.69	5.67	5.69	14.73	5.86	112.96	30.33	0.30	0.35	0.80
**M**	142924.73	13760.08	15337.49	8136.56	485.43	18.86	266.07	36.06	4.56	6.06	13.56	5.41	91.41	26.61	0.28	0.27	0.72
**SD**	20683.33	2281.43	2612.57	1254.20	46.65	2.71	52.27	5.10	1.02	1.04	1.75	0.59	19.40	3.77	0.03	0.06	0.10

ID	Na	Mg	K	Ca	Fe	Zn	B	Mn	Mo	Cu	Ni	Ba	Sr	Cr	As	Cd	Pb
ST15	158532.88	5915.28	23519.04	5875.10	345.68	36.79	0.00	11.38	1.29	13.57	2.46	6.45	46.68	0.91	0.33	0.15	0.93
ST15	236144.55	8273.06	33551.25	8517.10	455.24	45.65	0.00	16.00	1.62	17.86	3.21	8.31	66.24	1.24	0.31	0.19	1.14
ST15	187222.10	7143.53	29740.10	7399.54	401.54	42.08	0.00	14.09	1.46	15.94	3.00	7.17	58.39	1.13	0.29	0.15	0.96
**M**	193966.51	7110.62	28936.80	7263.91	400.82	41.50	0.00	13.82	1.46	15.79	2.89	7.31	57.10	1.10	0.31	0.16	1.01
**SD**	39242.94	1179.23	5064.12	1326.21	54.78	4.46	0.00	2.32	0.17	2.15	0.39	0.94	9.85	0.17	0.02	0.02	0.11
ST16	146137.72	22176.45	17352.68	22952.96	909.47	35.43	0.00	88.94	2.88	13.17	1.80	15.51	152.80	2.20	0.64	0.48	3.44
ST16	117023.84	19546.51	13928.27	25369.06	978.83	35.24	0.00	96.95	2.25	182.33	2.17	13.95	137.55	2.60	0.65	0.44	3.46
ST16	129628.76	22053.01	14518.76	29768.51	1126.47	33.61	0.00	106.94	2.38	16.30	2.14	16.50	150.19	2.84	0.76	0.46	3.48
**M**	130930.10	21258.66	15266.57	26030.18	1004.92	34.76	0.00	97.61	2.50	70.60	2.04	15.32	146.85	2.54	0.68	0.46	3.46
**SD**	14600.50	1484.05	1830.59	3455.54	110.83	1.00	0.00	9.02	0.33	96.77	0.21	1.29	8.15	0.32	0.06	0.02	0.02
ST17	176441.08	10857.19	22454.00	7313.85	145.53	14.25	0.00	25.62	2.08	6.39	1.76	2.11	56.97	2.32	0.11	0.07	0.25
ST17	202046.32	11054.41	21478.14	6928.59	138.69	21.70	0.00	27.67	1.91	6.23	0.82	1.94	52.94	0.33	0.16	0.06	0.22
ST17	162106.72	9445.76	20107.88	6491.58	117.51	15.07	0.00	22.63	1.63	5.61	0.70	1.62	48.09	0.32	0.12	0.06	0.23
**M**	180198.04	10452.45	21346.67	6911.34	133.91	17.01	0.00	25.30	1.87	6.08	1.10	1.89	52.67	0.99	0.13	0.06	0.23
**SD**	20233.12	877.38	1178.57	411.40	14.61	4.08	0.00	2.53	0.22	0.41	0.58	0.25	4.45	1.15	0.03	0.00	0.02

Table S3. ICP-MS (ppm) of succulent stems of *A. meridionale* (triplicates). Symbology: M (mean) and SD (Standard deviation). Samples nomenclature referred in Table S1.

ID	Na	Mg	K	Ca	Fe	Zn	B	Mn	Mo	Cu	Ni	Ba	Sr	Cr	As	Cd	Pb
ST18	91870.29	18609.34	11060.23	22526.94	1774.68	17.65	0.00	80.88	3.33	4.54	15.73	22.02	384.70	33.32	0.66	0.68	1.15
ST18	95514.15	21372.31	13782.56	26671.38	1867.40	19.86	0.00	92.31	3.80	5.54	14.70	19.61	461.71	31.60	0.62	0.73	1.16
ST18	100353.43	21682.79	14972.16	27024.30	1857.93	19.79	0.00	95.34	4.10	5.53	15.93	19.99	468.71	34.79	0.63	0.70	1.20
**M**	95912.62	20554.81	13271.65	25407.54	1833.34	19.10	0.00	89.51	3.75	5.20	15.46	20.54	438.38	33.24	0.63	0.70	1.17
**SD**	4255.59	1691.97	2005.38	2500.91	51.02	1.25	0.00	7.63	0.39	0.58	0.66	1.30	46.61	1.59	0.02	0.03	0.03
ST19	49147.93	21158.18	5147.32	11509.97	542.98	62.81	0.00	35.37	1.56	10.71	8.25	10.84	164.44	15.51	0.34	0.89	0.98
ST19	43019.97	18216.90	4200.22	9835.58	464.10	51.96	0.00	30.05	0.95	8.85	6.88	10.17	134.06	13.47	0.30	0.78	0.88
ST19	55658.49	25418.71	5570.38	13940.68	623.26	69.47	0.00	41.69	1.32	12.10	9.42	12.73	187.95	18.69	0.39	1.03	1.16
**M**	49275.46	21597.93	4972.64	11762.08	543.45	61.41	0.00	35.70	1.28	10.55	8.19	11.25	162.15	15.89	0.35	0.90	1.01
**SD**	6320.22	3620.99	701.58	2064.13	79.58	8.84	0.00	5.83	0.30	1.63	1.27	1.33	27.02	2.63	0.05	0.13	0.14
ST20	183799.33	11178.88	32058.68	9000.00	211.52	38.85	0.00	12.34	3.36	3.97	4.02	2.42	55.70	5.00	0.22	0.11	0.23
ST20	235235.97	14178.63	40843.19	11779.38	267.29	48.52	0.00	15.68	4.24	5.08	5.32	3.31	72.49	6.34	0.25	0.13	0.35
ST20	237456.82	13929.85	37923.72	11538.06	265.00	46.37	0.00	15.62	3.98	4.74	5.62	3.05	70.48	6.44	0.27	0.13	0.35
**M**	218830.71	13095.79	36941.86	10772.48	247.93	44.58	0.00	14.55	3.86	4.60	4.99	2.93	66.22	5.93	0.25	0.12	0.31
**SD**	30358.38	1664.74	4473.81	1539.75	31.56	5.08	0.00	1.91	0.45	0.57	0.85	0.46	9.17	0.80	0.03	0.01	0.07
